# Provision of group psychoeducation for relatives of persons in inpatient depression treatment – a cross-sectional survey of acute care hospitals in Germany

**DOI:** 10.1186/1471-244X-14-143

**Published:** 2014-05-19

**Authors:** Fabian Frank, Christine Rummel-Kluge, Mathias Berger, Eva M Bitzer, Lars P Hölzel

**Affiliations:** 1 Department of Psychiatry and Psychotherapy, University Medical Center Freiburg, Hauptstraße 5, D-79104 Freiburg, Germany; 2 Department of Public Health and Health Education, University of Education Freiburg, Kunzenweg 21, D-79117 Freiburg, Germany; 3 Department of Psychiatry and Psychotherapy, University Medical Center Leipzig, Semmelweisstraße 10, D-04103 Leipzig, Germany

**Keywords:** Psychoeducation, Relatives, Family, Depressive disorders, Inpatient treatment, Germany

## Abstract

**Background:**

Depressive disorders are often recurrent and place a high burden on patients and their relatives. Psychoeducational groups for relatives may reduce relatives’ burden, help prevent relapses in patients, and are recommended by the German “National Disease Management Guideline Unipolar Depression”. Since there is limited knowledge on the provision of psychoeducational groups for relatives of persons in inpatient depression treatment, we conducted a survey among acute care hospitals in Germany.

**Methods:**

We conducted a two-step cross-sectional survey. Step I consisted of a questionnaire asking the heads of all psychiatric/psychosomatic acute care hospitals in Germany (N = 512) whether psychoeducational groups for relatives were provided within depression treatment, and if not, the reasons for not implementing them. In group offering hospitals the person responsible for conducting psychoeducational groups received a detailed questionnaire on intervention characteristics (step II). We performed descriptive data analysis.

**Results:**

The response rate was 50.2% (N = 257) in step I and 58.4% in step II (N = 45). 35.4% of the responding hospitals offered psychoeducational groups for relatives of patients with depressive disorders. According to the estimates of the respondents, relatives of about one in five patients took part in psychoeducational groups in 2011. Groups were mostly provided by two moderators (62.2%) as continuous groups (77.8%), without patients’ participation (77.8%), with up to ten participants (65.9%), consisting of four or fewer sessions (51.5%) which lasted between one and one and a half hours each (77.8%). The moderators in charge were mostly psychologists (43.9%) or physicians (26.8%). Approximately one third used published manuals. Reasons for not conducting such psychoeducational groups were lack of manpower (60.1%), time (44.9%) and financial constraints (24.1%). 25.3% mentioned adequate concepts of intervention as a required condition for initiating such groups.

**Conclusions:**

Only a small proportion of relatives of patients with depressive disorders participated in psychoeducational groups in 2011 in Germany. Mostly short interventions were favoured and main implementation barriers were scarce resources. Brief interventions that fit with healthcare routine should be developed and tested within randomised controlled trials. This could promote the provision of psychoeducational groups for relatives as evidence-based practice in inpatient depression treatment in Germany.

## Background

Depressive disorders are highly prevalent [1] and one of the leading causes of years lived with disability worldwide [2]. They have a severe effect on psychosocial functioning [3] as well as the family life of the patients [4]. Due to the patients’ mood disturbance and illness-related behaviour, relatives of patients with depressive disorders experience heavy psychosocial burden [5]. The relatives’ burden is also rooted for example, in financial constraints due to the illness [6], increased household responsibilities, as well as limitations in social activities, social relationships or strains in the partnership [7,8]. Uncertainties about how to deal adequately with the patients and their illness-related behaviour [9] as well as negative emotions like guilt, anger or worries about the patients’ future are common [8]. A lack of illness-specific information and education, coupled with a high subjective need in this regard and a perceived lack of involvement in depression treatment, is also experienced as a burden [8,10,11]. Consequences are a diminished quality of life [12,13] and a significantly increased prevalence of depressive disorders in relatives themselves [14].

In Germany, patients with depressive disorders are frequently referred to inpatient treatment [15,16]. Although inpatient depression treatment is effective, relapses constitute one of the main problems [17] and rehospitalisations are common within the first year after discharge [16]. Research has shown a correlation between the burden of relatives and relapse in patients with severe mental illnesses [18,19]. Moreover, an association between a high level of expressed emotion and burden of relatives has been shown [20,21], as well as a relationship between a high level of expressed emotion and relapse in patients with depressive disorders [22].

For schizophrenia, *psychoeducational group interventions for relatives* (PGIR), which consider the information needs and burden of relatives as well as expressed emotion, are recommended in the current German treatment guidelines as an important part of the treatment [23], as they are suitable for reducing relapse and rehospitalisations [24,25], decreasing the burden of relatives and improving family functioning [26]. Also for depressive disorders, a recent Japanese study by Shimazu et al. [27] showed a significantly lower relapse rate among patients whose relatives took part in short PGIR (consisting of four group sessions addressing ‘epidemiology and causes’, ‘symptoms’, ‘treatment and course’, and ‘coping with the patient’, and group discussions for problem-solving) compared to patients whose relatives did not receive any intervention. Additionally, there are indications that short PGIR are likely to reduce emotional distress, care burden, expressed emotion and depressive symptoms among relatives of patients with depressive disorders [28]. In Germany, randomised controlled trials examining the efficacy of PGIR for patients with depressive disorders as well as their relatives are so far lacking.

However, the German “National Disease Management Guideline Unipolar Depression” recommends PGIR as a sensible addition to depression treatment with a grade of recommendation “B” as a “should do recommendation” [17]. However, meaningful data about the health care situation regarding PGIR in inpatient depression treatment in Germany as well as potential implementation barriers are largely missing, and little is known about how PGIR are conducted in routine health care. Knowledge about the manner in which PGIR are conducted in routine health care could contribute to the development of interventions feasible in everyday clinical practice. In light of this, the aim of the study was to examine to what extent inpatient depression treatment facilities in Germany provide PGIR, how they conduct PGIR, and if not, why not. Therefore, we conducted a two-part postal cross-sectional study in all hospitals providing acute care inpatient depression treatment in Germany.

## Methods

### Study sample

Acute care inpatient depression treatment in Germany takes place in specialised hospitals for psychiatry or psychosomatic medicine, respectively, in departments for psychiatry or psychosomatic medicine at general hospitals as well as university medical centres [17]. All corresponding hospitals and departments in Germany were included. We excluded specific centres, e.g. hospitals for geriatric psychiatry or child and adolescent psychiatry. Based on the *German Hospital Inventory* (Verzeichnis der Krankenhäuser und Vorsorge- oder Rehabilitationseinrichtungen in Deutschland) of the *Federal Statistical Office*[29], N = 512 hospitals Germany-wide met the inclusion criteria and were eligible for this study.

### Design and questionnaires

Following a design used in a study about psychoeducation in schizophrenia in inpatient treatment in Germany [30], a two-step postal cross-sectional study using a two-part questionnaire was conducted between July and November 2012. The first step of the study aimed to determine:

• characteristics of the participating hospitals (e.g. number of beds; hospital type);

• whether PGIR were actually offered in depression treatment in these hospitals;

• if not, reasons for not conducting PGIR and required conditions for initiating PGIR;

• if so, the contact person responsible for conducting PGIR in the particular hospital.

The second step aimed to obtain detailed information about how PGIR were conducted, specifically in consideration of:

• structural framework conditions (e.g. form and onset of groups; number, duration and frequency of sessions; number of participants; number and profession of the moderators);

• standardisation of the interventions (e.g. using a manual; using standardised material);

• aims, content and didactic methods used within the interventions;

• estimated percentage of patients whose relatives took part in PGIR in 2011.

Step I included a self-report questionnaire with nine questions, which was sent to the medical directors of all hospitals which met the eligibility criteria. Step II included a self-report questionnaire with 29 questions sent to the persons responsible for conducting PGIR according to step I.

Both questionnaires consisted of closed multiple-choice questions with and without the possibility for the participants to add their own answers, filter questions as well as open-ended questions. The work of Rummel-Kluge et al. [30] and Friedl-Huber et al. [31] forms the basis for the questionnaires. The questionnaires were administered in German language and are available upon request from the first author.

In both steps, questionnaires were provided with a cover letter in which psychoeducation was defined according to the widely recognised definition of the German expert group *Psychoeducational interventions for schizophrenic disorders* (Arbeitsgruppe Psychoedukation bei schizophrenen Erkrankungen) as “systematic, structured, didactic information on the illness and its treatment, which includes integrating emotional aspects in order to enable the participants to cope with the illness” [32]. The letter stated that the questionnaires refer to PGIR for relatives of adult patients with depressive disorders. In both steps, we informed the addressed persons about the possibility to return the questionnaires by post, fax, or scanned and sent by email. We sent reminders (including the questionnaire) to the nonresponders, six weeks after the initial letter, as these measures are associated with a better response rate [33].

To check for selection bias, we selected a random 25% sample of the hospitals, which did not respond to the step I questionnaire three months after the reminder (nonresponder-sample), and assessed by telephone whether or not they were offering PGIR in depression treatment in this sample.

### Ethical considerations

We submitted the study protocol to the Ethics Review Committee of the Albert-Ludwigs-University Freiburg, Germany. The study was approved as not required to be audited, because it is purely epidemiological and only health care-specific data regarding PGIR in inpatient depression treatment should be collected. The purpose and procedures of the study as well as data protection were explained in the cover letters of both questionnaires.

### Statistical analysis

We conducted descriptive data analysis by calculating frequencies as well as mean scores and standard deviations, modes and median using *IBM SPSS Statistics for Windows*, version 20.

## Results

### Step I

The response rate for step I was 50.2% (N = 257 of N = 512 hospitals). Characteristics of the participating hospitals are summarised in Table 1. With an average of 130 inpatient beds, these hospitals served an average of about 500 patients with depressive disorders in 2011.

**Table 1:** Hospital characteristics

**Hospital size**		**Inpatient vs. day clinic (n = 257)**^**1**^	
number of inpatient beds^2,3^ (n = 228)	129.8 (115.0)	solely inpatient	16.3
≤ 50 beds^1^	18.9	inpatient and day clinic	72.8
51 – 100 beds^1^	30.7	solely day clinic	10.9
101 – 150 beds^1^	21.0	**hospital type (n = 255)**^**1**^	
≥ 151 beds^1^	29.4	specialised hospital	48.6
patients with depression treated in 2011^2^ (n = 219)	486.9 (457.8)	dept. general hospital	35.7
≤ 150 patients^1^	24.2	dept. university med. centre	9.0
151 – 300 patients^1^	16.4	others	6.7
301 – 450 patients^1^	20.1	**hospital orientation (n = 257)**^**1**^	
451 – 600 patients^1^	13.3	psychiatric	76.3
≥ 601 patients^1^	26.0	psychosomatic	16.3
number of day clinic places^2,4^ (n = 214)	32.3 (22.6)	not specified	7.4

^1^in percent.

^2^means and standard deviation.

^3^only hospitals with inpatient beds.

^4^only hospitals with day clinic places.

Overall – during the time of the survey – 35.4% of the respondents report that PGIR were conducted as part of depression treatment in their hospitals. 51.6% of the PGIR offering hospitals offer depressive-specific PGIR, 40.7% offer PGIR combining depressive disorders with other diagnostic groups (mostly ICD-10 F2: 80.6% (N = 25); other F3 diagnoses: 61.3% (N = 19); F4: 32.3% (N = 10); F6: 32.3% (N = 10); others: 16.1% (N = 5); sum greater than 100% due to multiple response options; total N = 31) and 7.7% offer specific PGIR as well as combined PGIR. 64.6% of the respondents state that PGIR were not conducted at all as part of depression treatment in their hospitals. Reasons were mostly lack of resources (manpower, time, finances) but also lacking concepts of intervention for PGIR in depression treatment (for details, cf. Table 2). When respondents not offering PGIR in depression treatment were asked for required conditions for initiating PGIR, the most frequently stated answers were additional resources (additional staff; more time and financial resources) as well as adequate concepts of intervention (for details, cf. Table 2).

**Table 2:** Reasons for not conducting PGIR and required conditions for initiating PGIR

**Reasons for not conducting PGIR**^**1 **^**(n = 158)**			
lack of manpower	60.1	too few patients with depression	5.1
lack of time	44.9	PGIR considered as irrelevant	3.2
financial constraints	24.1	PGIR not considered as hospital’s task	2.5
lacking concepts of intervention	15.2	others*	40.5
hospitalisation too short	7.6	*in particular: relatives are involved otherwise, catchment area too big, low acceptance of the intervention	
**Required conditions for initiating PGIR**^**1 **^**(n = 158)**			
additional staff	67.1	none, PGIR are considered as irrelevant	4.4
more time	49.4	none, PGIR are not considered as hospital’s task	1.3
adequate concepts of intervention	25.3	others*	22.2
more financial resources	24.1	*in particular: bigger catchment area, higher acceptance of the intervention, bigger hospital	

^1^in percent; sums greater than 100% due to multiple response options.

Of those hospitals randomly chosen for the nonresponder-sample (N = 64), 7.8% (N = 5) gave no information or were not accessible. 16.9% (N = 10) of the hospitals which participated in the nonresponder analysis (N = 59) stated that they did offer PGIR in depression treatment.

### Step II

Of the N = 91 respondents which stated that they offered PGIR in depression treatment in step I, 84.6% (N = 77) named a contact person responsible for offering PGIR. N = 45 of the step II questionnaires sent to these contact persons were returned, with the response rate for step II lying at 58.4%. Thus, detailed data about how PGIR were conducted were gathered from 49.5% of all hospitals, which stated that they offered PGIR in depression treatment in step I.

Most of the respondents in step II were psychologists (40.9%), 31.8% were physicians, 15.9% social workers and 11.4% were nurses. 91.1% of the respondents worked at a hospital with a psychiatric orientation. 42.2% were employed at a specialised hospital for mental illnesses, 33.3% at a general hospital, 20.0% at a university medical centre and 4.4% at another hospital type. All of the results presented below refer solely to hospitals participating in step II.

Of those hospitals conducting PGIR in depression treatment and responding to step II, 77.8% offered PGIR without participation of the patient and 22.2% offered PGIR in which patients and relatives took part in the same group. Overall, the respondents estimated a mean of about 18% (SD 16%; Mode 20%; Median 12.5%; Range 1% - 70%) of patients with depressive disorders whose relatives took part in PGIR in 2011. Respondents stated that a mean of about 29% (SD 16%) of the relatives taking part in PGIR discontinued their participation. Reasons for discontinuation included termination of the group sessions, lack of time and health burden of the relatives themselves.

Standardised manuals are used in more than half of the PGIR (24.4% completely manualised; 40.0% partly manualised). Of those respondents using standardised manuals (N = 29), about one half use self-developed and the other half use published manuals [34-37]; therefore, published manuals are at least partly used by about one third of respondents. 75.6% of the respondents use standardised information material, in particular handouts, presentations as well as brochures (for details, cf. Table 3). The most frequently mentioned didactic methods within the PGIR are discussions and lectures (for details, cf. Table 3).

**Table 3:** Standardised information material and didactic methods used

**Standardised materials**^**1**^** (n = 45)**			
utilisation of standardised material	75.6		
if standardised material is used (n = 34)			
handouts	61.8	slides	35.3
presentations	44.1	flip chart	26.5
brochures	41.2	videos/DVDs	14.7
**Didactic methods**^**1 **^**(n = 45)**			
discussion	95.6	role play	20.0
lecture	88.9	behavioural training	13.3
small groups	22.2	others	20.0

^1^in percent; sums greater than 100% due to multiple response options

Study participants were also asked how they advertised their PGIR (sums add up to more than 100% because of multiple response options). 75.6% of the respondents stated that they invite relatives indirectly through the patients; 44.4% of the hospitals invite relatives via personal contact, 26.7% invite relatives directly via written invitation, and 64.4% invite relatives upon request to participate in the PGIR. Furthermore, PGIR are announced via flyers (55.6%) and posters (53.3%), but other methods of distribution are also chosen (31.1%), in particular via the internet and advertisements in the local press.

Regarding the structure (for details, cf. Table 4), PGIR is mostly conducted in an open or continuous form (71.1%). The onset of participation in the PGIR is mostly independent of inpatient depression treatment (57.8%). 51.5% of the hospitals offer PGIR with four or fewer group sessions. Group sessions are held usually weekly (35.6%) and last one to one and a half hour (77.8%). The typical group size is six to ten relatives. The majority of participating relatives are the patients’ partners (53.5%), 28.6% are parents, 10.8% are patients’ children and 7.1% are others, in particular siblings and friends as well as in rare cases parents-in-law, grandparents or neighbours.

**Table 4:** Structure of the PGIR

**Form of groups**^**1 **^**(n = 44)**		**Duration of a session in minutes**^**1**^** (n = 45)**	
closed	15.6	≤ 60	6.7
partially closed	13.3	61-90	77.8
open or continuous	71.1	≥ 91	15.6
**Onset of groups**^**1 **^**(n = 44)**		**Frequency of sessions**^**1 **^**(n = 45)**	
during inpatient treatment only	15.6	weekly	35.6
continuation after discharge	24.4	bi-weekly	17.8
during outpatient treatment only	2.2	monthly	26.7
independent of inpatient treatment	57.8	other frequencies	20.0
**Number of sessions**^**1 **^**(n = 33)**		**Average number of participants**^**1 **^**(n = 41)**	
≤ 4	51.5	≤ 5	17.1
5-8	36.4	6-10	48.8
≥ 9	12.1	11-15	22.0
		≥ 16	12.2

^1^in percent.

In two thirds of the PGIR two group moderators are present (62.2%), one group moderator only is involved in 28.9% of the PGIR. A few respondents state they use three or four moderators (8.9%). 75.6% of the moderators or co-moderators are physicians, 66.7% psychologists, 42.2% nursing staff, 40.0% social workers and in rare cases also clinical pastoral staff, occupational therapists or other relatives (sums add up to more than 100% due to multiple response options). Primarily the person in charge or the responsible moderator for conducting PGIR, respectively, is a psychologist (43.9%); 26.8% are physicians, 17.1% social workers and 12.2% nursing staff.

In an open-ended question, respondents were asked about the goals they pursued within the PGIR and their responses were subsequently categorised (sums add up to more than 100% due to multiple answers). The most frequently addressed **goals within the PGIR** are: to improve self-care and relief strategies in relatives (68.2%), to inform relatives about the illness, its symptoms and causes as well as its treatment (59.1%), to foster the relatives’ ability to deal adequately with the illness and support the patient (52.3%), and to create a better understanding of the illness and the patient in relatives (47.7%). Further goals are to stabilise the family climate (27.3%), to enhance communication between relatives and patients (20.5%), relapse prevention and a better compliance of the patient (11.4%), and to facilitate inter-exchange between relatives (9.1%). 6.8% stated early recognition of warning signs as being a goal of the PGIR. The most frequently addressed **information topics within the PGIR** are how to deal adequately with the patient (90.9%) as well as relief strategies in order to reduce caregiver burden (86.4%). However, other behaviour-related topics (e.g. communication patterns) and illness-specific (e.g. symptoms and diagnoses) as well as treatment-related information contents (e.g. pharmacotherapy) are also addressed frequently (for details, cf. Table 5). The most frequently addressed **emotional topics within the PGIR** are excessive demands (95.6%) and helplessness on the part of the relatives (91.1%) as well as suicidality of the patient (82.2%) and feelings of guilt and shame (77.8%) of the relatives (for details, cf. Table 5).

**Table 5:** Frequency of information and emotional topics addressed in PGIR

**Frequency of information topics**^**1,2 **^**(n = 44)**		
1	dealing adequately with the patient	90.9%
2	decompression strategies	86.4%
3	symptoms and diagnosis	75.0%
4	communication patterns	61.4%
	contingency plan	61.4%
5	warning signs	59.1%
6	pharmacotherapy	54.5%
7	psychotherapy	52.3%
	risk factors	52.3%
8	cause of illness	50.0%
9	relapse prevention	47.7%
10	sociotherapy	40.9%
11	course of illness	36.4%
12	problem solving	31.8%
13	epidemiology	9.1%
**Frequency of emotional topics**^**1 **^**(n = 45)**		
1	excessive demands	95.6
2	helplessness	91.1
3	suicidality	82.2
4	guilt and shame	77.8
5	partnership	75.6
6	stigmatisation	71.1
7	isolation	60.0
8	quarrel with destiny	46.7
	resignation	46.7
	anergy	46.7

^1^in percent; sums greater than 100% due to multiple response options.

^2^out of six possible categories (“1 = none” to “6 = very much”), categories five and six were added and ranked according to their frequency.

In addition to the frequency of information and emotional topics, respondents were asked about their experience regarding which topics relatives needed to discuss most during the PGIR within an open-ended question, which was subsequently categorised (sums add up to more than 100% due to multiple answers). In accordance with the information and emotional topics which were addressed most frequently, the respondents saw the highest **need for discussion** on the part of relatives with regard to dealing adequately with the patient and the illness (56.8%), helplessness (22.7%), excessive demands (15.9%), feelings of guilt (15.9%), own needs and relief (13.6%), suicidality (11.4%), pharmacotherapy (11.4%), partnership (9.1%) and relapse prevention (6.8%).

Regarding **expressed emotion**, 83.7% of the respondents stated that the offered PGIR consisted of measures to reduce criticism and 82.9% of respondents stated measures to reduce emotional overinvolvement. When asked in an open-ended question to specify these measures, respondents cited information about the illness, behavioural and communication exercises regarding dealing with the patient, measures to improve self-care as well as explaining expressed emotion.

## Discussion

By means of a two-step cross-sectional survey, this study aimed to examine how and to what extent PGIR are provided in routine health care in inpatient depression treatment in Germany. In hospitals without PGIR implementation, barriers were explored. The results might contribute to a better understanding of how PGIR should be conceptualised to fit with routine health care in everyday clinical practice. In both steps, high response rates comparable to similar studies [30,38,39] were achieved (step I: 50.2%; step II 58.4%). However, as not all hospitals replied, the critical issue is the representativeness of this sample. It remains unclear, how much PGIR is being conducted in the nonresponding hospitals. This lack may have caused a bias of the results. Most probably an overestimation of the actual amount PGIR are offered, as reporting positive aspects more often than negative ones is well known. Therefore a nonresponder analysis was conducted.

35.4% of the responding hospitals offered PGIR in inpatient depression treatment. However, as only 16.9% of the responding hospitals of the nonresponder-sample provided PGIR, this finding seems to overestimate the real amount. Furthermore, it can be assumed that hospitals offering PGIR were more likely to take part in this study than hospitals without PGIR. As 35.4% (N = 91) of the responding hospitals (N = 257) offered PGIR, and assuming that the amount of 16.9% (N = 10) which was found in the responder population of the nonresponder-sample (N = 59) reflects the amount of PGIR offered in the whole nonresponding population (N = 255), it can be assumed that – despite current recommendations of German treatment guidelines for unipolar depression [17] – only about one quarter of all acute care hospitals in Germany offer PGIR in inpatient depression treatment. This reflects a difference between actual treatment recommendations and current practice in routine health care regarding the provision of PGIR in inpatient depression treatment.

If PGIR is conducted, about two thirds of the hospitals use at least partly manualised interventions, whereby about one half uses published and the other half uses self-developed manuals. Thus, only about one third of the hospitals providing PGIR use comparable and reproducible interventions, which fit with published recommendations for PGIR defined by several experts for PGIR in Germany – e.g. [32]. This finding suggests that the amount of PGIR which are conducted according to these recommendations is considerably lower, than the amount of hospitals offering PGIR in inpatient depression treatment in Germany.

However, if PGIR is conducted, they mostly seemed to be in accordance with widely recognised German manuals for PGIR in depressive disorders – e.g. Pitschel-Walz et al. [34]; Schaub et al. [35]; Wilms et al. [40] – regarding information contents and emotional topics to be addressed as well as most of the framework conditions. The most frequently addressed information topics are dealing adequately with the patient, decompression strategies as well as symptoms and diagnosis. Regarding emotional topics, excessive demands, helplessness, suicidality, feelings of guilt and shame as well as strains in the partnership are addressed most frequently. These contents are in accordance with findings about information needs [9,11] and burden of relatives [7,8]. From the PGIR-contact persons point of view, relatives’ most urgent need is dealing adequately with the patient. This can be considered as link to broaching the issue of expressed emotion – an important predictor of relapse in patients with depressive disorders [22]. From our step II survey a typical PGIR appears to be an open or continuous weekly group, with a size of 10 participants, targeting at relatives (no depressive patients), lasting one to one and half hour. However, as more than half of the PGIR consisted of four or fewer group sessions, they differed widely from the above-cited German manuals for PGIR in depressive disorders, which propose eight to twelve group sessions. This reveals a gap between routine health care and suggested German manualised PGIR for depression treatment. The higher use of shortened interventions might be explained by lacking resources: PGIR are often held in the evenings to ensure that working relatives can participate. This may lead to unpaid work after the regular working hours on the part of the moderators.

In those hospitals offering PGIR, according to respondents’ estimates from step II, relatives of about one in five patients with depressive disorders took part in PGIR in 2011. This is a higher proportion of participants than was found for PGIR in inpatient treatment in schizophrenia in Germany [30] as well as for educational or support programmes for relatives of patients with schizophrenia in the United States of America [41]. Thus, we assume that relatives of patients with depressive disorders are interested in participating in PGIR. However, this finding is susceptible to bias. For instance, as it is based on estimations of the respondents, and the study was not anonymised, the result might have been overestimated due to social desirability. As most responding hospitals did not offer PGIR, only a small proportion of relatives of patients with depressive disorders was considered in inpatient depression treatment in Germany in 2011.

These findings suggest that despite the recommendation of the German “National Disease Management Guideline Unipolar Depression” and possible positive effects of PGIR on patients and their relatives PGIR are not well established and only a small proportion of relatives participated in PGIR in 2011. On the other hand, when respondents without PGIR were asked about reasons for not conducting respective interventions, only less than 5% considered PGIR as irrelevant or not as their task. In accordance with the findings of other studies [30,42], the most frequently mentioned implementation barriers were lack of manpower, time and financial resources, and about one quarter stated that adequate concepts of intervention are required for initiating PGIR in their hospitals.

As PGIR are judged as helpful by relatives with respect to information regarding the illness and strategies for dealing with the patient [43], as they could reduce the burden of relatives [28] and contribute to a better course of illness in patients [27], are recommended by the current German treatment guidelines [17] and are seen as relevant by clinicians, possibilities to increase their provision should be found. With respect to scarce resources, the absence of an adequate financing basis for PGIR, the preference for short-term interventions in routine health care, as well as the stated lack of adequate concepts of intervention, the development and evaluation of shortened interventions could contribute to spreading the provision of PGIR in inpatient depression treatment in Germany. In accordance with public recommendations [32] as well as the findings of this study, the most important key content areas that should be focused on in the development of brief PGIR are behavioural contents like dealing with the patient, communication patterns and decompression strategies as well as educational contents like symptoms and diagnosis, treatment options for depressive disorders and cause of illness. A challenge concerning the development of brief PGIR will be to assure the quality and depth of these contents as well as a sufficient coverage for outlining issues regarding dealing with the patient and expressed emotion. Furthermore, in the development of such interventions, the stated requirement of additional staff for initiating PGIR should be considered by integrating the whole team to facilitate the implementation.

As only a small proportion of relatives of patients with depressive disorders are given the opportunity to benefit from PGIR, efforts have to be made to provide such interventions more frequently and more consistently across institutions and patients’ relatives. As maybe more hospitals would imply PGIR in inpatient depression treatment if it would be recognized as an evidence based method, future research should focus on (1.) improving the evidence for already existing PGIR as well as (2.) the development of evidence-based interventions, which take into consideration the scarce resources in the current German health care system and are feasible to routine health care, in order to improve the quality and quantity of health services with respect to the involvement of relatives. A promising approach regarding this matter was presented in a recent randomised controlled trial, conducted in Japan by Shimazu et al. [27]. In their study a brief PGIR consisting of four group sessions – addressing behavioural and educational topics in line with public German recommendations [32] as well as the results of our study – showed a high efficacy regarding relapses in patients after inpatient depression treatment. In an additional pilot study using a similar intervention, positive effects on expressed emotion and caregiver burden were shown [28]. However, this evidence is restricted to Japan. In view of differences in the health care systems as well as cultural differences between the two countries, the intervention cannot be directly transferred to Germany. Nevertheless, due to the encouraging results the cultural adaptation of the intervention seems promising. Such a cultural adaption should focus on the specific needs of relatives in Germany as well as differences in the understanding of depressive disorders, be in accordance with current German treatment guidelines for unipolar depression, consider differences in the health care systems and reflect cultural aspects regarding the didactic methods of choice as well as cultural differences in communication patterns.

## Conclusions

Based on the results of the present study it can be assumed that in 2011 only about one quarter of the acute care hospitals respectively departments for psychiatry or psychosomatic medicine in Germany provided PGIR in inpatient depression treatment. Relatives of only a small proportion of patients with depressive disorders participated in PGIR. PGIR is provided, mostly as brief intervention. Implementation barriers were mainly scarce resources but also a lack of interventions that fit with routine health care. The development and evaluation of manualised, brief PGIR which fit with routine health care and are feasible in everyday clinical practice should be undertaken to promote the provision of psychoeducational groups for relatives as evidence-based practice in inpatient depression treatment in Germany.

## Competing interests

The authors declare that they have no competing interests.

## Authors’ contributions

FF conceptualised and coordinated the study, participated in designing the study and the questionnaires, performed the acquisition of data and data management, performed the statistical analyses and wrote the paper. CRK participated in designing the study as well as the questionnaires and revised the paper. MB contributed to the study design and revised the paper for important intellectual content. EMB participated in the conceptualisation and design of the study as well as the questionnaires and edited various drafts of the paper. LPH participated in the conceptualisation and design of the study as well as the questionnaires, supported the acquisition of data, supported the statistical analyses and edited various drafts of the paper. EMB and LPH supervised the study. All authors read and approved the final manuscript.

## Pre-publication history

The pre-publication history for this paper can be accessed here:

http://www.biomedcentral.com/1471-244X/14/143/prepub
